# What are the factors associated with depressive symptoms among orphans and vulnerable children in Cambodia?

**DOI:** 10.1186/s12888-015-0576-9

**Published:** 2015-07-29

**Authors:** Ken Ing Cherng Ong, Siyan Yi, Sovannary Tuot, Pheak Chhoun, Akira Shibanuma, Junko Yasuoka, Masamine Jimba

**Affiliations:** 1 Department of Community and Global Health, Graduate School of Medicine, The University of Tokyo, 7-3-1 Hongo, Bunkyo-ku, Tokyo, 113-0033 Japan; 2 Center for Population Health, KHANA, Phnom Penh, Cambodia

**Keywords:** Orphans and vulnerable children (OVC), Depressive symptoms, Mental health, Cambodia

## Abstract

**Background:**

Compared to general children, orphans and vulnerable children (OVC) are more exposed to negative outcomes in life such as abuse and neglect. Consequently, OVC are more susceptible to depression. This paper investigated factors associated with depressive symptoms among OVC in Cambodia.

**Methods:**

In this cross-sectional study, data of 606 OVC from the Sustainable Action against HIV and AIDS in Communities (SAHACOM) project were analyzed. The data were collected from five provinces and analyzed separately for boys and girls. Multiple linear regression analysis was used to identify factors independently associated with levels of depressive symptoms.

**Results:**

Both boys and girls who reported having been too sick making them unable to attend school or go to work in the past six months (boys: B = 3.5; 95 % CI = 0.7, 6.2; girls: B = 5.7; 95 % CI = 2.9, 8.5) and who had witnessed violence in the family (boys: B = 5.6; 95 % CI = 1.6, 9.6; girls: B = 5.8; 95 % CI = 1.7, 9.9) had a higher level of depressive symptoms. Girls who were older (B = 8.5; 95 % CI = 3.0, 14.0), who did not have enough food in the past six months (B = −8.7; 95 % CI = −13.7, −3.7) and whose parents were separated, divorced or dead (B = 3.9; 95 % CI = 0.5, 7.2) had a higher level of depressive symptoms. Higher level of school attachment was negatively associated with depressive symptoms in both genders (boys: B = −1.4; 95 % CI = −2.0, −0.9; girls: B = −1.4; 95 % CI = −2.0,-0.9).

**Conclusions:**

Factors such as physical health and exposure to violence may affect mental health of OVC in Cambodia. As health is of utmost importance, better healthcare services should be made easily accessible for OVC. Schools have the potential to act as a buffer against depressive symptoms. Therefore, efforts should be made to keep OVC in school and to improve the roles of school in Cambodia.

## Background

Childhood is a pivotal period for a child’s overall development [1]. The survival and development of a child’s optimal potential are disrupted if the family environment is jeopardized due to illness or death of the parents or guardians [2]. The loss of parents or guardians might also cause withdrawal, anxiety, and depression in adolescence [2].

Adverse childhood experiences (ACEs) are stressful events such as physical abuse, emotional abuse, sexual abuse, parental separation or divorce, neglect, and violence in the family [3]. ACEs could leave a long-lasting adverse impact on a child’s mental development [4, 5]. In addition, stress response as a result of ACEs could also affect the neurobiological development of a child’s brain. When a child encounters a stressful experience such as abuse, the development of certain parts of the brain such as the hippocampus and the corpus callosum is hindered [6, 7]. Consequently, this may lead to the manifestation of psychiatric disorders such as schizophrenia, attention deficit hyperactivity disorder (ADHD), and unipolar and bipolar depression [6, 7]. Moreover, ACEs have been found to be associated with lower self-esteem in early adulthood, and lower well-being or clinical disorder later in life [8, 9]. In addition, ACEs have also been linked to early alcohol initiation and ever using alcohol during adolescence [10].

Orphans and vulnerable children (OVC) comprise orphans and children who are more exposed to detrimental events such as abuse, neglect and exploitation compared to their peers. OVC include street children, children made vulnerable by AIDS, children in the worst forms of child labor, children affected by armed conflict, children living with disability, and children in multiple OVC categories [11]. OVC are at a higher risk of ACEs such as physical abuse, emotional abuse, or sexual abuse than general children [12].

Furthermore, OVC might also be forced to shoulder the responsibility of an adult, while still coping with the trauma of losing a caregiver such as parents [13]. OVC have to take care of their younger siblings or become the sole breadwinner of their household [14]. Consequently, these OVC might be forced to suspend school or drop out of school to take care of their family [15]. OVC with extra workload were more likely to exhibit depressive symptoms [16]. Moreover, OVC are also more likely to face stigma and discrimination in the community [14].

Psychological distress might lead OVC to engage in risky sexual behaviors [17]. As a result, these OVC might be more susceptible to HIV infection, and their offspring might also become vulnerable; thus perpetuating the negative cycle. In addition, OVC also have a higher tendency to possess juvenile delinquent traits such as theft and poor socialization [18, 19].

Cambodia is a low income country in South East Asia with a population of 15.1 million and a GDP of $15.2 billion as of 2014 [20]. Several decades of war and conflict have shattered this South East Asian kingdom that was once the center of one of the greatest civilizations in the region. Similar to OVC in other parts of the world, OVC in Cambodia are more likely to be malnourished and to drop out of school and prone to depression [21].

Notwithstanding the situation in Cambodia, most studies on the psychological well-being among OVC were conducted in sub-Saharan Africa [14, 15, 17–19]. Moreover, different studies in different countries and cultures yielded different results. To illustrate this point, a study in South Africa showed that female OVC tended to have a higher level of depressive symptoms compared to male OVC [22]. In contrast, a study in Uganda found that male OVC were at a greater risk of depression compared to female OVC because girls tended to receive more nurturing and attention in the Ugandan context [23]. Therefore, OVC’s psychological response to their own situation is very contextual with the influence from each child’s unique background, surrounding, and gender.

In a previous study, several factors such as exposure to family and community violence were associated with depressive symptoms among adolescent students in Cambodia [24]. However, no studies have yet explored factors associated with the psychological well-being among OVC in the Cambodian context. We, therefore, conducted this study to fill in this knowledge gap to identify factors associated with depressive symptoms among OVC in Cambodia. Findings from this study would extend the understanding of the effects of social factors on psychological well-being in different adolescent populations in the country.

## Methods

This is a cross-sectional study, and data were collected in April and May 2014 as part of the impact evaluation of the Sustainable Action against HIV and AIDS in Communities (SAHACOM) project. The details of participants and sampling have been described elsewhere [25]. Briefly, data were collected from five provinces in Cambodia: Battambang, Pailin, Pursat, Siem Reap, and Takeo. These five provinces were chosen because more than 70 % of the total number of OVC covered by the SAHACOM project was from these provinces. The OVC were randomly selected from a name list of OVC who received care and support services from each selected health center in the five provinces [25].

For this study, only participants aged 11 and above were included in the final analysis as depressive symptoms were only assessed among participants in this age group. After excluding OVC with missing responses to key variables, the final sample size for the analysis was 606.

### Socio-demographic characteristics

Demographic variables in this study included age, self-reported HIV status, orphan status, sibling care practices, food security, and general health status. For age, participants were categorized into four groups: 11–12, 13–14, 15–16, and 17–18. For the self-reported HIV status, participants were grouped into three categories: HIV positive, HIV negative, and don’t know. Regarding the orphan status, we grouped the participants into non-orphans, single orphans, and double orphans. To assess sibling care practices, we asked the participants whether they had to take care of siblings or relatives younger than five years in the past 12 months. Concerning food security, we inquired the participants whether they had enough food to eat in the past six months prior to the interview. As for general health status, we asked the participants whether they had been very sick making them unable to work or go to school the past six months.

### Depressive symptoms

The main variable is depressive symptoms, and it was measured using the Asian Adolescent Depression Scale (AADS). This scale contains 20 items measuring four dimensions: negative self-evaluation (seven items), negative affect (five items), cognitive inefficiency (four items), and lack of motivation (four items) [26]. This scale provides a five-point Likert’s response options ranging from (1) ‘Strongly disagree’ to (5) ‘Strongly agree.’ The total score is the sum of the 20 items ranging from 20 to 100. Higher level of depressive symptoms is indicated by a higher score. This scale has been used to measure depressive symptoms among Cambodian adolescents in other studies [24, 27, 28]. The Cronbach’s α for this study was 0.87.

### Adverse childhood experiences

For ACEs, five questions were adapted from the brief screening version of the Childhood Trauma Questionnaire [29, 30]. The five questions asked about the experience of physical abuse, emotional abuse, sexual abuse, physical neglect, and emotional neglect. The response options for each question range from (1) ‘never’ to (5) ‘very often.’ Participants who responded ‘never’ and ‘rarely’ were grouped together as those without ACEs. Participants who answered ‘sometimes,’ ‘often’, and ‘very often’ were grouped together as those with ACEs.

### Family dysfunction

Five items adapted from the brief screening version of the Childhood Trauma Questionnaire were used to enquire about family dysfunction [29, 30]. These five items asked about ‘witnessing violence against a family member,’ ‘having an alcoholic or drug user family member,’ ‘having a family member who was depressed, mentally ill or who has attempted suicide,’ ‘having parents who had been separated or divorced,’ and ‘having a family member who has been to prison.’ The response options for all the items were ‘yes’ or ‘no,’ except for ‘having parents who had been separated or divorced.’ For this item, another response option was added to the ‘yes’ or ‘no’ responses to indicate if one or both parents had died. For the analysis in this study, participants whose parents had divorced or separated were grouped together with participants whose parent/s had died.

### School attachment

School attachment was measured using a seven-item scale adapted from a previous study [31]. These seven items were: ‘I like school,’ ‘My teachers like me,’ ‘I like my teachers,’ ‘School is fun,’ ‘I am accepted in school,’ ‘I feel like an outsider in school,’ and ‘I feel like I fit in at school.’ These seven items were measured on a 4-point scale that includes (0) ‘not at all,’ (1) ‘not much,’ (2) ‘some’ and (3) ‘a lot.’ The response to ‘I feel like an outsider in school’ was reverse coded before obtaining the total score for all seven items. Better school attachment is indicated by a higher score. This scale had been used in previous studies among Cambodian adolescents [24, 27]. The Cronbach’s α for this study was 0.56.

### Data analyses

The total scores for the AADS and School Attachment Scale were calculated. To address gender differences, analyses were conducted separately for boys and girls [24, 32, 33]. For bivariate analyses, one-way analysis of variance (ANOVA) or *t*-test was used.

In the multiple linear regression models, variables were included when they were found to be associated with depressive symptoms in previous studies. These variables were age, HIV status, orphan status, sibling care practices, food security, general health status, ACEs, family dysfunction, and school attachment [23, 28, 34–36].

Multicollinearity was not a concern in the models. This conclusion was reached by obtaining the variance inflation factor (VIF) values for all the variables after running the regression. All the variables had the VIF values less than 2.0, which were far below 10.0, the recommended VIF value to further examine the data for multicollinearity [37]. All tests were two-tailed and statistical significance was set at *p* < 0.05. Stata Version 12.1 (College Station, Texas, USA) was used for all analyses.

### Ethical considerations

KHANA obtained the ethical approval from the National Ethics Committee for Health Research, Ministry of Health, Cambodia, for the data collection (No. 082NECHR). This study protocol was reviewed and approved by the Research Ethics Committee of the University of Tokyo (No. 10723). Privacy of the participants was strictly protected as no personally identifying information was used in this study. In addition, the participants were ensured the confidentiality of their responses to allay their fear when answering sensitive questions such as physical abuse, sexual abuse, and family dysfunction. A written informed consent was also obtained from the OVC’s parent or guardian.

## Results

There were 606 participants in this study, of whom 303 (50.0 %) were boys. All participants were 11–18 years old at the time of data collection, and the mean age was 13.4 years (SD 1.7). Regarding the HIV status, 471 (77.7 %) participants reported that they were HIV negative, 116 (19.1 %) were HIV positive and 19 (3.1 %) were not sure about their HIV status. Concerning the orphan status, 360 (59.4 %) OVC were non-orphans, while 182 (30.0 %) and 64 (10.6 %) were single orphans, and double orphans, respectively. The majority of boys (97.3 %) and girls (97.3 %) were currently in school.

Table 1 shows the comparisons of mean AADS score in socio-demographic characteristics stratified by gender. Both boys and girls who reported having been too sick making them unable to attend school or go to work in the past six months had a significantly higher mean AADS score (*p* = 0.008 for boys & *p* < 0.001 for girls). However, only girls who were in the older age group (*p* = 0.002), who had to regularly take care of younger siblings (*p* = 0.018), and who did not have enough food to eat in the past six months had a significantly higher mean AADS score (*p* < 0.001).

**Table 1:** Comparisons of mean AADS score in socio-demographic characteristics stratified by gender

		Boys			Girls		
		n (%)	Mean	*p*-value	n (%)	Mean	*p*-value
Age							
	11-12	102 (33.7)	46.2	0.534	90 (29.7)	43.8	0.002
	13-14	138 (45.5)	47.9		116 (38.3)	46.9	
	15-16	49 (16.2)	47.4		76 (25.1)	45.6	
	17-18	14 (4.6)	50.3		21 (6.9)	55.5	
Self-reported HIV Status							
	Positive	64 (21.1)	48.3	0.763	52 (17.2)	46.2	0.479
	Negative	231 (76.2)	47.1		240 (79.2)	46.5	
	Don’t know	8 (2.6)	47.8		11 (3.6)	41.6	
Orphan Status							
	Non-orphan	184 (60.7)	47.9	0.392	176 (58.1)	46.2	0.076
	Single orphan	93 (30.7)	47.1		89 (29.4)	44.6	
	Double orphan	26 (8.6)	44.6		38 (12.5)	50.3	
Regularly taken care of siblings and younger relatives younger than five years	Yes	103 (34.0)	48.1	0.471	106 (35.0)	48.6	0.018
	No	200 (66.0)	47.0		197 (65.0)	45.0	
Have enough food to eat in the past 6 months	Yes	286 (94.4)	47.2	0.239	279 (92.1)	45.3	<0.001
	No	17 (5.6)	50.6		24 (7.9)	57.3	
Have been very sick in the past 6 months	Yes	113 (37.3)	49.7	0.008	106 (35.0)	50.2	<0.001
	No	190 (62.7)	46.0		197 (65.0)	44.1	

*AADS* Asian Adolescent Depression Scale

Table 2 shows the comparisons of mean AADS score in ACEs and family dysfunction stratified by gender. Experiences of all forms of ACEs were not significantly associated with the mean AADS score in boys. However, mean AADS score was significantly higher among girls who had been physically abused (53.8 *vs.* 45.5; *p* = 0.001), emotionally abused (51.4 *vs.* 45.2; *p* = 0.002), and emotionally neglected (56.6 *vs.* 46.0; *p* = 0.021). Both boys and girls who had witnessed violence in the family had a significantly higher mean AADS score (51.9 *vs.* 46.6; *p* = 0.007 for boys & 53.1 *vs.* 45.3; *p* < 0.001 for girls). However, only among girls, OVC who had a family member who used alcohol or illicit drugs (49.2 *vs.* 45.5; *p* = 0.045), who had a depressed or mentally ill family member (52.7 *vs.* 45.2; *p* < 0.001), and whose parents had separated, divorced or died (47.5 *vs.* 44.5; *p* = 0.047) had a significantly higher mean AADS score.

**Table 2:** Comparisons of mean AADS score in adverse childhood experiences and family dysfunction stratified by gender

		Boys			Girls		
		n (%)	Mean	*p*-value	n (%)	Mean	*p*-value
Physical abuse	Yes	46 (15.2)	47.4	0.990	28 (9.2)	53.8	0.001
	No	257 (84.8)	47.4		275 (90.8)	45.5	
Emotional abuse	Yes	55 (18.2)	48.8	0.327	52 (17.2)	51.4	0.002
	No	248 (81.8)	47.1		251 (82.8)	45.2	
Sexual abuse	Yes	6 (2.0)	52.2	0.316	3 (1.0)	43.7	0.728
	No	297 (98.0)	47.3		300 (99.0)	46.3	
Physical neglect	Yes	9 (3.0)	47.3	0.993	7 (2.3)	48.9	0.589
	No	294 (97.0)	47.4		296 (97.7)	46.2	
Emotional neglect	Yes	8 (2.6)	46.4	0.810	8 (2.6)	56.6	0.021
	No	295 (97.4)	47.4		295 (97.4)	46.0	
Witnessed violence in family	Yes	42 (13.9)	51.9	0.007	37 (12.2)	53.1	<0.001
	No	261 (86.1)	46.6		266 (87.8)	45.3	
Had an alcoholic/drug using family member	Yes	65 (21.5)	48.1	0.577	61 (20.1)	49.2	0.045
	No	238 (78.5)	47.2		242 (79.9)	45.5	
Had depressed or mentally ill family member	Yes	32 (10.6)	44.5	0.152	43 (14.2)	52.7	<0.001
	No	271 (89.4)	47.7		260 (85.8)	45.2	
Had a family member who had been to prison	Yes	15 (5.0)	45.9	0.631	14 (4.6)	52.8	0.052
	No	288 (95.0)	47.4		289 (95.4)	45.9	
Parents separated/divorced or died	Yes	161 (53.1)	47.4	0.946	176 (58.1)	47.5	0.047
	No	142 (46.9)	47.3		127 (41.9)	44.5	

*AADS* Asian Adolescent Depression Scale

Table 3 shows factors associated with depressive symptoms after controlling for other factors in the models. Having been too sick making them unable to attend school or go to work in the past six months (boys: B = 3.5; 95 % CI = 0.7, 6.2; girls: B = 5.7; 95 % CI = 2.9, 8.5) and having witnessed violence in the family (boys: B = 5.6; 95 % CI = 1.6, 9.6; girls: B = 5.8; 95 % CI = 1.7, 9.9) were positively associated with a higher level of depressive symptoms among both boys and girls. Having a mentally ill family member was negatively associated with a higher level of depressive symptoms only among boys (B = −5.5; 95 % CI = −9.9, −1.1). Among girls, being in the older age group (B = 8.5; 95 % CI = 3.0, 14.0), and having parents who were separated, divorced, or dead (B = 3.9; 95 % CI = 0.5, 7.2) were positively associated with a higher level of depressive symptoms. Having enough food to eat in the past six months (B = −8.7; 95 % CI = −13.7, −3.7) was negatively associated with a higher level of depressive symptoms only among girls. Having a higher level of school attachment was negatively associated with a higher level of depressive symptoms for both boys (B = −1.4; 95 % CI = −2.0, −0.9) and girls (B = −1.4; 95 % CI = −2.0,-0.9).

**Table 3:** Factors associated with depressive symptoms among orphans and vulnerable children in Cambodia

	Boys			Girls		
	B	*p*-value	95 % CI	B	*p*-value	95 % CI
Age (*vs.* 11–12)						
13−14	2.7	0.074	(−0.3, 5.7)	2.2	0.161	(−0.9, 5.3)
15−16	3.1	0.125	(−0.9, 7.1)	2.1	0.247	(−1.5, 5.6)
17−18	1.6	0.629	(−5.0, 8.2)	8.5	0.003	(3.0, 14.0)
HIV Status (*vs.* HIV positive)						
Negative	−1.0	0.525	(−4.2, 2.2)	1.4	0.430	(−2.1, 4.9)
Don’t know	2.2	0.608	(−6.3, 10.8)	−1.8	0.638	(−9.2, 5.6)
Orphan status (*vs.* non−orphans)						
Single orphans	−1.6	0.370	(−5.2, 1.9)	−3.4	0.059	(−7.0, 0.1)
Double orphans	−2.3	0.409	(−7.8, 3.2)	−0.5	0.834	(−5.0, 4.1)
Having to take care of younger siblings/relatives	−1.2	0.399	(−4.1, 1.6)	2.1	0.120	(−0.5, 4.8)
Having been too sick in the past 6 months	3.5	0.014	(0.7, 6.2)	5.7	<0.001	(2.9, 8.5)
Having enough food to eat	−2.0	0.504	(−8.0, 3.9)	−8.7	0.001	(−13.7, −3.7)
Physical abuse	−0.8	0.688	(−4.6, 3.1)	4.0	0.091	(−0.6, 8.6)
Emotional abuse	1.5	0.388	(−1.9, 4.9)	2.1	0.254	(−1.5, 5.7)
Sexual abuse	2.3	0.629	(−7.2, 11.8)	−0.7	0.909	(−13.7, 12.2)
Physical neglect	1.1	0.775	(−6.7, 8.9)	3.9	0.395	(−5.1, 12.9)
Emotional neglect	3.8	0.358	(−4.3, 12.0)	0.2	0.969	(−8.3, 8.6)
Witnessed violence in family	5.6	0.006	(1.6, 9.6)	5.8	0.006	(1.7, 9.9)
Had an alcoholic/a drug using family member	−2.2	0.213	(−5.8, 1.3)	1.0	0.541	(−2.3, 4.4)
Had a mentally ill family member	−5.5	0.014	(−9.9, −1.1)	1.4	0.493	(−2.6, 5.3)
Separated or divorced or dead parents	1.1	0.540	(−2.3, 4.4)	3.9	0.026	(0.5, 7.2)
Had a family member who had been to prison	1.5	0.631	(−4.7, 7.7)	5.6	0.075	(−0.6, 11.7)
School attachment	−1.4	<0.001	(−2.0, −0.9)	−1.4	<0.001	(−2.0, −0.9)

*CI* confidence interval

## Discussion

This study adds to the literature on factors associated with depressive symptoms among OVC in Cambodia. Both boys and girls who reported having been too sick making them unable to work or go to school in the past six months had a higher level of depressive symptoms. In Cambodia, infectious diseases such as acute respiratory infections and malaria still affect the general population including the younger generation [38]. These diseases cause a loss in productivity and prolonged illness among those affected [39]. Moreover, injuries from traffic accidents have also become a major health issue among young people in Cambodia [38, 40]. Injuries from traffic accidents are a major concern as they could cause permanent loss in productivity. Prolonged illness might cause a sense of despair among the affected OVC as they had to cope with both the demands of the illness and their daily chores. On top of that, being very sick would affect the OVC’s school attendance, and this in turn would affect the OVC’s psychological well-being. A study in Zimbabwe reported that a higher prevalence of psychological distress was more common among OVC who were out of school [41].

In the family where a guardian was physically hurt by another family member, both boys and girls had a significantly higher level of depressive symptoms. However, a different study in Cambodia reported that no significant association was detected between family violence witnessing and depressive symptoms among boys and girls [24]. The contrasting results in this study might be because witnessing violence on another family member aggravates the psychological burden already present among OVC. This would leave the OVC in constant fear of being harmed themselves. Exposure to violence during childhood has been found to be an independent risk factor for depressive symptoms in early adulthood [42]. By the same token, children’s exposure to violence could result in internalizing and externalizing behavior problems during adolescence [43].

Boys who had a mentally ill family member reported a significantly lower level of depressive symptoms. OVC usually have to shoulder extra responsibilities such as caring for other ill family member [16]. However, it is very demanding to care for other family members, especially those with mental illness. This could result in resentment and distress among the caregivers [44]. Although very demanding, caring for other mentally ill family members has positive effects on the caregiver as the caregiver might feel a sense of fulfillment and perceived uplift [45]. Furthermore, compared to female caregivers, male caregivers were found to have a better style of managing mentally ill persons under their care and thus, were able to detach themselves from stressful situations resulting from their responsibility [44]. The sense of fulfillment and perceived uplift, and a better style of managing mentally ill persons among male caregivers might explain why boys with a mentally ill family member had a significantly lower level of depressive symptoms.

In this study, older age, lack of food, and parents’ separation or death resulted in a significantly higher level of depressive symptoms among girls. However, boys in the same categories did not exhibit a significantly higher level of depressive symptoms. More women are affected by depression compared to men [33]. Higher level of depressive symptoms among girls could be accounted for by considering the status of women in the Cambodian context. Girls might be facing constant pressure to conform to societal norms. According to a traditional Khmer proverb, ‘Men are gold; women are cloth,’ women are considered vulnerable and fragile and once defamed, the damage to their reputation could not be undone [46]. In households with OVC, boys are still given preference over girls when it comes to providing education in resource-limited settings [47].

When parents of girls were separated, divorced or dead, the girls exhibited a significantly higher level of depressive symptoms. This may be because girls are more likely to exhibit internalizing symptoms such as withdrawal, anxiety, and depression in stressful situations [48]. The same reasoning could also be used to account for the higher level of depressive symptoms among girls who did not have enough food to eat. In this study, 35.0 % of the girls had to take care of a younger sibling or relatives. As these girls were entrusted with the task of caring for younger siblings or relatives, the lack of food to feed their younger charges might also be a cause of stress and depression.

In this study, HIV status and orphan status were not significantly associated with depressive symptoms. The reason might be because the majority of OVC in this study benefited from the support provided by the SAHACOM project. This is evident in the improvement of the key indicators at the end of SAHACOM project such as an increase in school attendance and also child care support [25, 49].

Higher school attachment was found to have a positive effect on mental health of OVC in this study. Boys and girls with a higher level of school attachment had a significantly lower level of depressive symptoms. The role of school transcends that of a place that provides only academic guidance for the OVC [50]. School is a place where OVC can find comfort and connect with their peers. OVC who attended school regularly were also found to have a higher level of perceived social support and in turn, exhibited lower degree of depression and higher self-esteem [15].

Findings in this study should, however, be interpreted in light of several limitations. First, the cross-sectional design of this study did not allow us to infer the causal relationship between depressive symptoms and the related factors. Second, the use of self-reported data in this study might be subject to recall bias. However, to ensure the quality of the data collected, all the interviewers and field supervisors were thoroughly trained on the data collection method before going to the field [25]. Third, some of the measures such as ACEs and family dysfunction were adapted from previous studies, and have not been validated in the Cambodian context. Finally, the types of sickness among the OVC were not determined. Hence, future studies should also attempt to identify the types of sickness among the OVC to help create a more efficient healthcare service for the OVC in Cambodia. Notwithstanding the limitations above, findings from this study have strengths and implications for policy development and future research. To the best of our knowledge, this is the first study to explore the factors associated with mental health wellbeing among OVC in the Cambodian context.

## Conclusion

This study highlighted important factors associated with depressive symptoms among OVC in Cambodia. As evident from the results of this study, there are both similar and different factors affecting depressive symptoms among boys and girls. As physical health is of utmost importance for both boys and girls for survival, more comprehensive healthcare services should be made easily accessible for OVC all over the country. In addition to that, healthcare workers should also be trained to identify depressive symptoms, especially for girls as they are more susceptible to depression. Furthermore, schools have the potential to act as a protective factor against depression among OVC. Therefore, more efforts should be channeled into improving the role of schools in Cambodia. Teachers should be trained to distinguish children with depressive symptoms and pay more attention to children who are identified as OVC. Future research might also attempt to focus on factors associated with depression among subgroups of OVC such as maternal orphans, children living with HIV or children affected by HIV. Additionally, future interventions might also focus on empowering OVC by developing their perceived control of the future. A study conducted in China indicated that positive future orientation and hopefulness could alleviate the adverse effects of traumatic events among OVC [51]. This might give OVC hope and motivation for the future and help them chart out their own life; one where they could live with dignity.
